# Incorporating inflammatory biomarkers into a prognostic risk score in patients with non-ischemic heart failure: a machine learning approach

**DOI:** 10.3389/fimmu.2023.1228018

**Published:** 2023-08-15

**Authors:** Jiayu Feng, Xuemei Zhao, Boping Huang, Liyan Huang, Yihang Wu, Jing Wang, Jingyuan Guan, Xinqing Li, Yuhui Zhang, Jian Zhang

**Affiliations:** ^1^ State Key Laboratory of Cardiovascular Disease, Heart Failure Center, National Center for Cardiovascular Diseases, Fuwai Hospital, Chinese Academy of Medical Sciences and Peking Union Medical College, Beijing, China; ^2^ Key Laboratory of Clinical Research for Cardiovascular Medications, National Health Committee, Beijing, China

**Keywords:** biomarker, inflammation, non-ischemic heart failure, predictive model, machine learning

## Abstract

**Objectives:**

Inflammation is involved in the mechanisms of non-ischemic heart failure (NIHF). We aimed to investigate the prognostic value of 21 inflammatory biomarkers and construct a biomarker risk score to improve risk prediction for patients with NIHF.

**Methods:**

Patients diagnosed with NIHF without infection during hospitalization were included. The primary outcome was defined as all-cause mortality and heart transplantations. We used elastic net Cox regression with cross-validation to select inflammatory biomarkers and construct the best biomarker risk score model. Discrimination, calibration, and reclassification were evaluated to assess the predictive value of the biomarker risk score.

**Results:**

Of 1,250 patients included (median age, 53 years, 31.9% women), 436 patients (34.9%) experienced the primary outcome during a median of 2.8 years of follow-up. The final biomarker risk score included high-sensitivity C-reactive protein-to-albumin ratio (CAR) and red blood cell distribution width-standard deviation (RDW-SD), both of which were 100% selected in 1,000 times cross-validation folds. Incorporating the biomarker risk score into the best basic model improved the discrimination (Δ*C*-index = 0.012, 95% CI 0.003–0.018) and reclassification (IDI, 2.3%, 95% CI 0.7%–4.9%; NRI, 17.3% 95% CI 6.4%–32.3%) in risk identification. In the cross-validation sets, the mean time-dependent AUC ranged from 0.670 to 0.724 for the biomarker risk score and 0.705 to 0.804 for the basic model with a biomarker risk score, from 1 to 8 years. In multivariable Cox regression, the biomarker risk score was independently associated with the outcome in patients with NIHF (HR 1.76, 95% CI 1.49–2.08, *p* < 0.001, per 1 score increase).

**Conclusions:**

An inflammatory biomarker-derived risk score significantly improved prognosis prediction and risk stratification, providing potential individualized therapeutic targets for NIHF patients.

## Graphical Abstract

We utilized a machine learning-based elastic net Cox regression for variable selection from easily obtainable inflammatory biomarkers. We performed inner loop cross-validations to determine the parameter *λ* and then selected the model with the highest *C*-statistic in outer loops. The process above was repeated 1,000 times to choose the inflammatory markers that presented at 100% frequency in repetitions. We constructed a biomarker risk score based on CAR and RDW-SD. The biomarker risk score increased discrimination and reclassification of the predictive model.
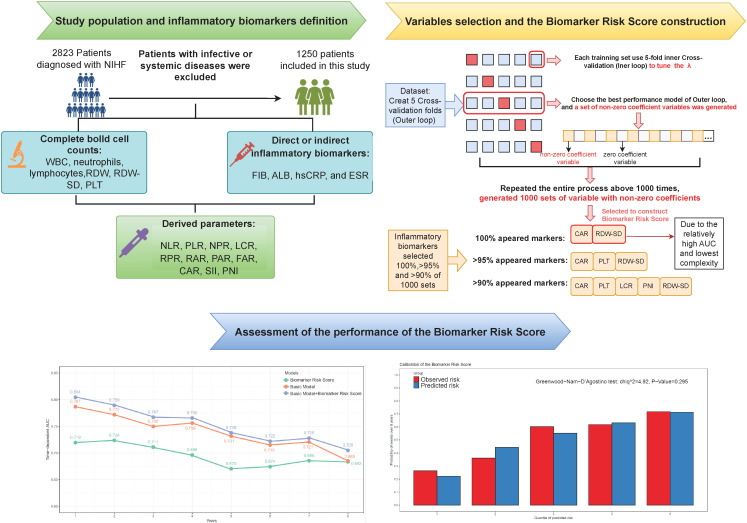

## 1 Introduction

Non-ischemic heart failure (NIHF) is a clinical syndrome with symptoms and/or signs, accompanied by elevated natriuretic peptide levels and/or objective evidence of congestion, in the absence of significant coronary artery disease (CAD) (1). This condition is associated with high mortality and requirement for heart transplantation. Despite advances in medical therapy, response to treatment can be variable, underscoring the need for accurate risk prediction and personalized management to improve outcomes.

Inflammation has been identified as a critical underlying mechanism in the development and progression of HF (2). Research has shown that NIHF is associated with a unique and persistent inflammatory response that differs from the acute myocardial ischemia and subsequent reperfusion injury seen in ischemic heart failure (3–5). These differences have significant implications for disease pathogenesis and treatment strategies. Inflammatory biomarkers, such as high-sensitivity C-reactive protein (hsCRP), fibrinogen (FIB), albumin, erythrocyte sedimentation rate (ESR), and indices within the complete blood cell count (including white blood cells [WBC], neutrophils, lymphocytes, and red blood cell distribution width [RDW]), have been identified as potential predictors of adverse outcomes in patients with CAD or HF (6–10). It is worth noting that, despite biomarkers like FIB, albumin and RDW were not traditionally used as inflammatory biomarkers; several studies have shown that these biomarkers can reflect chronic inflammation, and inflammatory activation may be the central link in the prognostic role of these biomarkers (6, 11). Furthermore, derived parameters from these biomarkers, such as the fibrinogen-to-albumin ratio (FAR), hsCRP-to-albumin (CAR), neutrophil-to-lymphocyte ratio (NLR), systemic immune-inflammation index (SII), and prognostic nutritional index (PNI), have also been demonstrated to serve as prognostic factors (11–14). However, the prognostic value of inflammatory biomarkers in NIHF and which parameters are the most predictive remain largely unknown.

Our study aimed to use a machine learning approach to investigate and identify the most predictive inflammatory biomarkers and their derived parameters for the prognosis of NIHF. Additionally, we aimed to develop a biomarker risk score that incorporates these valuable indexes to enhance the accuracy of NIHF risk prediction.

## 2 Materials and methods

### 2.1 Patients

This study retrospectively included patients who were diagnosed with NIHF and aged >18 years old, between 2006 and 2017, at the Heart Failure Care Unit of Fuwai Hospital. The diagnosis of NIHF was made based on clinical presentation and objective evidence, such as imaging or natriuretic peptides, in the absence of significant CAD (myocardial infarction [MI], stent implantation, or coronary artery bypass grafting, ≥50% stenosis confirmed by CTA or coronary angiography). Patients with infective or systemic diseases were excluded from the study, including those with (1) viral myocarditis, (2) infective endocarditis, (3) cancer, (4) autoimmune disease, (5) blood system disease, and (6) infection during hospitalization. Ethical approval was obtained from the Ethics Committee of Fuwai Hospital, and all participants provided written informed consent (Approval number 2014-501).

### 2.2 Follow-up and endpoint

During the follow-up period, the participants were given suitable medical treatment as directed by the guideline. The composite outcome was established as the combination of all-cause mortality and heart transplantation because these events can serve as hard endpoints to reflect the prognosis of NIHF patients.

### 2.3 Data collection and inflammatory parameters definition

The study obtained information on demography, symptoms and signs, laboratory examination, therapies, and echocardiography from the Fuwai Electronic Medical Record System. A vein blood sample was collected from all hospitalized patients on the morning following admission and collected in EDTA tubes. All biomarkers were tested at the central laboratory following standard procedures. The inflammatory biomarkers analyzed in this study were comprehensively evaluated using both single and derived parameters. We investigated 10 single parameters (white blood cell [WBC], neutrophils, lymphocytes, red blood cell distribution width [RDW], RDW-SD, platelet [PLT], fibrinogen [FIB], albumin, high-sensitivity C-reactive protein [hsCRP], and erythrocyte sedimentation rate [ESR]) and 11 derived parameters, namely, neutrophil-to-lymphocyte ratio (NLR), platelet-to-lymphocyte ratio (PLR), neutrophil-to-platelet ratio (NPR), lymphocyte-to-hsCRP ratio (LCR), RDW-to-platelet ratio (RPR), RDW-to-albumin ratio (RAR), platelet-to-albumin ratio (PAR), FIB-to-albumin ratio (FAR), hsCRP-to-albumin ratio (CAR), SII (neutrophil * platelet/lymphocyte), and PNI (albumin+5 * lymphocyte).

### 2.4 Statistical analysis

#### 2.4.1 Baseline characteristics demonstration

Baseline characteristics are presented as frequencies (percentages) for categorical variables and medians (25th to 75th percentile) for continuous variables. Characteristics were compared using a *χ* ^2^ test or the Fisher exact test for categorical variables and a Student *t*-test or Mann–Whitney *U*-test for continuous variables.

#### 2.4.2 Inflammatory biomarker selection and the biomarker risk score construction

For inflammatory variable selection and risk score construction, we utilized a machine learning-based elastic net Cox regression that combines Ridge (L2) and LASSO (L1) regularization. This approach was chosen because it can help to mitigate the impact of multicollinearity and to identify the most important variables (15). All inflammatory markers were standardized to *z*-scores (mean = 0, standard deviation [SD] = 1) prior to input. The relative contribution of L1 and L2 regularization is controlled by a mixing parameter *α*, which was set to 0.5. The elastic net Cox regression was performed by the R package “glmnet”. To determine the optimal value of the model complexity parameter *λ*, we performed a fivefold cross-validation inner loop and selected the *λ* value that resulted in the minimum partial likelihood deviation. Next, the best *λ* value obtained from the inner loop was used to fit a model in each training set of a fivefold outer loop cross-validation. For inflammatory biomarker selection, we then selected the model with the highest *C*-statistic in the corresponding test set of the outer loop, and the best model in each outer loop produced a set of inflammatory biomarkers with non-zero coefficients.

To generate a stable model with the most effective variables, we repeated the entire process above 1,000 times and chose the inflammatory biomarkers that presented at 100% frequency in repetitions to construct the biomarker risk score. The coefficients of the variables included in the biomarker risk score were determined by fitting them into a new elastic net Cox regression. To compare different variable selection strategies, we tested the performance of models constructed using biomarkers selected at >95% and >90% frequency of the 1,000 cross-validation iterations, compared to the model with 100% appeared variables.

#### 2.4.3 The basic predictive model construction

The basic model was constructed using the same approach of elastic net Cox regression incorporating age, gender, systolic blood pressure (SBP), New York Heart Association (NYHA) III/IV, current smoking, dilated cardiomyopathy (DCM), chronic obstructive pulmonary disease (COPD), atrial fibrillation (AF), diabetes, N-terminal Pro Brain natriuretic peptide (NT-proBNP), serum creatine (Scr), hemoglobin, low-density lipoprotein cholesterol (LDL-C), therapy with angiotensin-converting enzyme inhibitor/angiotensin receptor blocker (ACEI/ARB), and beta-blockers.

#### 2.4.4 Assessment of the performance of the biomarker risk score and the biomarker risk score plus basic model

Regarding discrimination, we evaluated the time-dependent receiver operating characteristic (ROC) area under the curve (AUC) of the biomarker risk score from 1 to 8 years. The time-dependent ROC was performed by the R package “timeROC”. To test the model’s stability, we also presented the mean and SD of the time-dependent AUC in 100 times fivefold cross-validation. The improvement in the Harrel’s *C*-statistic (Δ*C*-index) by adding the biomarker risk score to the basic model was also assessed. We tested the 95% confidence interval (CI) of the Δ*C*-index in 1,000 bootstrap samples. To assess calibration, we used the Greenwood–Nam–D’Agostino (GND) test to evaluate the agreement between observed and predicted risk, where *p* < 0.05 indicated lack of fit. For reclassification assessment, we conducted continuous net reclassification improvement (NRI) and integrated discrimination improvement (IDI) analyses at 8 years. The IDI and NRI were calculated by the R package “survIDINRI”. Lastly, we performed Cox regressions to investigate the independent prognostic roles of the biomarker risk score and its components after adjusting for covariates in the basic model. The Schoenfeld residual was used to test the proportional hazard assumption by the R function “coxzph”. We reported the hazard ratio (HR) and 95% CI, and considered *p* < 0.05 to be statistically significant. We conducted all statistical analyses using R software version 4.1.3.

## 3 Results

### 3.1 Baseline characteristics

This study included 1,250 hospitalized patients diagnosed with NIHF (**Supplementary Figure 1**). **Table 1** provides a summary of baseline characteristics based on the primary outcome. The median age of the patients was 53 years (interquartile range, 42–64), with 339 (31.9%) being women. Patients who met the endpoint had higher levels of RDW, RDW-SD, ESR, and hsCRP, while having lower levels of lymphocyte, PLT, and albumin than patients who did not meet the endpoint. We also examined derived parameters, finding that patients who met the endpoint had higher levels of FAR, CAR, RAR, RPR, NLR, PLR, and NPR, while having lower levels of PAR, LCR, and PNI (**Supplementary Table 1**).

**Table 1:** Baseline characteristics for NIHF patients with or without primary outcome.

	Overall	Primary outcome (−)	Primary outcome (+)	*p*-value
*N*	1,250	814	436	
Clinical characteristics				
Age (years)	53 [42, 64]	52 [41, 63]	56 [45, 66]	0.001
Female (%)	399 (31.9)	258 (31.7)	141 (32.3)	0.866
Heart rate (b.p.m.)	80 [69, 93]	80 [70, 94.8]	79 [68, 90]	0.037
SBP (mmHg)	116 [102, 130]	120 [107, 133]	106 [96, 120]	<0.001
DBP (mmHg)	70 [62, 80]	72 [65, 81.8]	68 [60, 75]	<0.001
BMI (kg/m^2^)	24.2 [21.3, 27.1]	24.8 [22.0, 27.7]	22.8 [20.5, 25.9]	<0.001
DCM (%)	510 (40.8)	326 (40.0)	184 (42.2)	0.498
Valvular heart disease (%)	160 (12.8)	100 (12.3)	60 (13.8)	0.512
T2DM (%)	200 (16.0)	133 (16.3)	67 (15.4)	0.714
COPD (%)	56 (4.5)	22 (2.7)	34 (7.8)	<0.001
Hypertension (%)	497 (39.8)	352 (43.2)	145 (33.3)	0.001
AF (%)	463 (37.0)	283 (34.8)	180 (41.3)	0.027
NYHA Class III/IV (%)	946 (75.7)	566 (69.5)	380 (87.2)	<0.001
Smoking (%)	283 (22.6)	178 (21.9)	105 (24.1)	0.412
Drinking (%)	253 (20.2)	173 (21.3)	80 (18.3)	0.253
Laboratory Test				
Fibrinogen (g/L)	3.39 [2.90, 4.06]	3.35 [2.90, 3.98]	3.45 [2.87, 4.16]	0.297
Albumin (g/L)	41.00 [37.50, 44.20]	41.70 [38.20, 44.80]	39.90 [36.50, 42.80]	<0.001
HsCRP (mg/L)	2.88 [1.41, 6.88]	2.49 [1.26, 5.56]	3.61 [1.71, 9.19]	<0.001
WBC (10^9^/L)	6.70 [5.52, 8.08]	6.72 [5.64, 8.12]	6.64 [5.15, 8.00]	0.092
Neutrophil (10^9^/L)	4.22 [3.34, 5.36]	4.16 [3.37, 5.30]	4.30 [3.28, 5.45]	0.796
Lymphocyte (10^9^/L)	1.72 [1.29, 2.20]	1.79 [1.40, 2.25]	1.51 [1.06, 2.05]	<0.001
Platelet (10^9^/L)	187.50 [148.00, 237.00]	194.00 [154.00, 244.00]	176.50 [138.00, 221.00]	<0.001
RDW (%)	13.70 [12.80, 14.90]	13.35 [12.60, 14.38]	14.45 [13.40, 15.70]	<0.001
RDW-SD (fl)	44.80 [41.60, 48.70]	43.75 [40.90, 46.80]	47.40 [43.30, 51.40]	<0.001
ESR (mm/h)	6.00 [2.00, 14.00]	6.00 [2.00, 12.00]	7.00 [3.00, 17.00]	<0.001
Hemoglobin (g/L)	143.0 [128.0, 157.0]	145.0 [131.0, 159.0]	138.5 [124.0, 153.0]	<0.001
Scr (μmol/L)	90.0 [74.6, 109.8]	87.4 [73.4, 106.1]	93.9 [77.4, 116.0]	<0.001
NT-Pro BNP (pg/ml)	1,939.8 [828.5, 4,101.5]	1,533.0 [658.5, 3,299.5]	3,070.2 [1,362.2, 5,601.5]	<0.001
**Echocardiography**	1.8 [1.6, 2.0]	1.8 [1.7, 2.0]	1.7 [1.6, 1.9]	<0.001
LAD (mm)	46.0 [41.0, 52.0]	45.0 [40.0, 50.0]	49.0 [44.0, 55.0]	<0.001
LVEDD (mm)	64.0 [53.0, 72.0]	63.0 [54.0, 70.0]	66.0 [52.2, 76.0]	0.005
LVEF (%)	35.6 [27.1, 53.0]	38.0 [29.0, 54.0]	32.0 [25.0, 51.0]	<0.001
RVD (mm)	24.0 [21.0, 28.0]	24.0 [21.0, 27.0]	26.0 [22.0, 31.0]	<0.001
Therapy				
Digoxin (%)	793 (63.4)	535 (65.7)	258 (59.2)	0.026
ACEI/ARB (%)	726 (58.1)	532 (65.4)	194 (44.5)	<0.001
β-blocker (%)	1,013 (81.0)	691 (84.9)	322 (73.9)	<0.001
MRA (%)	927 (74.2)	620 (76.2)	307 (70.4)	0.032
Diuretics (%)	1,041 (83.3)	674 (82.8)	367 (84.2)	0.589

Values are shown as median [interquartile range] or as frequencies [percentage]. SBP, systolic blood pressure; DBP, diastolic blood pressure; BMI, body mass index; DCM, dilated cardiomyopathy; T2DM, Type 2 diabetes mellitus; COPD, chronic obstructive pulmonary disease; AF, atrial fibrillation; NYHA, New York Heart Association; hsCRP, high-sensitivity C-reactive protein; WBC, white blood cell; RDW-SD, red blood cell distribution width-standard deviation; ESR, erythrocyte sedimentation rate; Scr, serum creatine; NT-Pro BNP, N-terminal Pro Brain natriuretic peptide; LAD, left atrial diameter; LVEDD, left ventricular end-diastolic diameter; LVEF, left ventricular ejection fraction; RVD, right ventricular diameter; ACEI, angiotensin-converting enzyme inhibitor; ARB, angiotensin receptor blocker; MRA, mineralocorticoid receptor antagonist.

### 3.2 Selection of inflammatory biomarkers for the biomarker risk score construction

**Figure 1:**
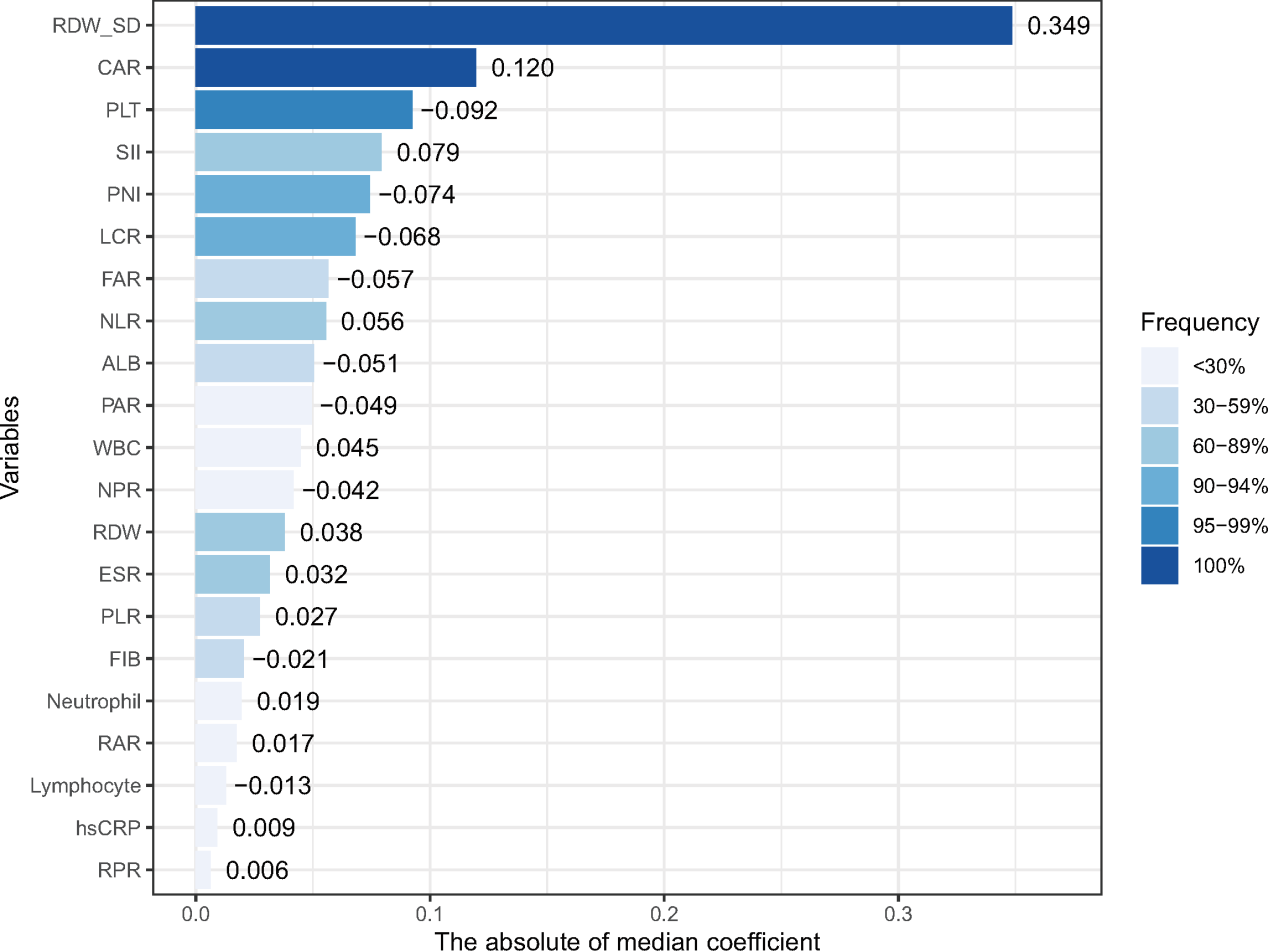
The frequencies of selection or each inflammatory marker in 1,000 cross-validations and their median coefficients. RDW-SD, red blood cell distribution width-standard deviation; PLT, platelet; FIB, fibrinogen; ALB, albumin; hsCRP, high-sensitivity C-reactive protein; WBC, white blood cell; ESR, erythrocyte sedimentation rate; NLR, neutrophil-to-lymphocyte ratio; PLR, platelet-to-lymphocyte ratio; NPR, neutrophil-to-platelet ratio; LCR, lymphocyte-to-hsCRP ratio; RPR, RDW-to-platelet ratio; RAR, RDW-to-albumin ratio; PAR, platelet-to-albumin ratio; FAR, FIB-to-albumin ratio; CAR, hsCRP-to-albumin ratio; SII, systematic inflammatory index (neutrophil * platelet/lymphocyte); and PNI, prognostic nutritional index (albumin+5 * lymphocyte).

According to our pre-defined inflammatory biomarker selection strategy, from 1,000 iterations of fivefold cross-validation, CAR and RDW-SD appeared in 100% of the 1,000 repetitions. Moreover, variables with a frequency >90% in the final model are CAR, LCR, PLT, PNI, and RDW-SD; those with a frequency >95% are CAR, PLT, and RDW-SD. The frequencies of selection and their median coefficients for each inflammatory biomarker are shown in **Figure 1**. Three models were constructed based on variables with frequencies 100%, >95%, and >90%, and their average *C*-index and average partial likelihood deviance in cross-validation are presented in **Supplementary Table 2**. We ultimately selected the variables with a frequency of 100% (CAR and RDW-SD) to construct the biomarker risk score, as this model had the lowest complexity and no significant difference in mean *C*-index, time-dependent AUC, and partial likelihood deviance compared to the other two models (**Supplementary Figure 2**). The linear predictor of elastic net regression including CAR and RDW-SD was calculated as the biomarker risk score in the total population, and the formula was biomarker risk score = 0.20*CAR+0.47*RDW-SD.

### 3.3 Predictive value of the biomarker risk score and adding the biomarker risk score to the basic model

**Figure 2:**
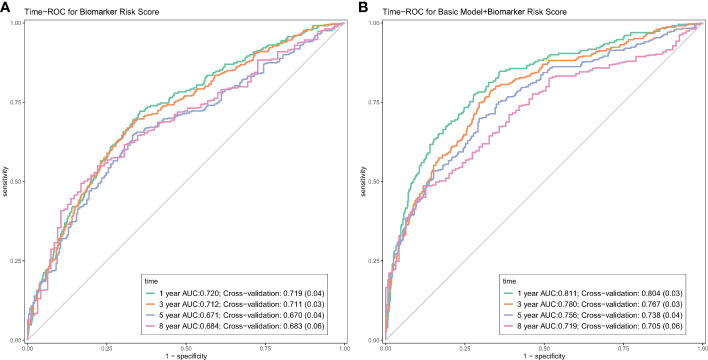
The time-dependent receiver-operating characteristic curves (ROC) of the biomarker risk score **(A)**, and the biomarker risk score plus basic model **(B)**. The basic model was also constructed using elastic net Cox regression incorporating age, gender, SBP, NYHA III/IV, current smoking, DCM, COPD, AF, diabetes, NT-proBNP, creatine, hemoglobin, LDL-C, therapy with ACEI/ARB, and beta-blockers.

**Figure 3:**
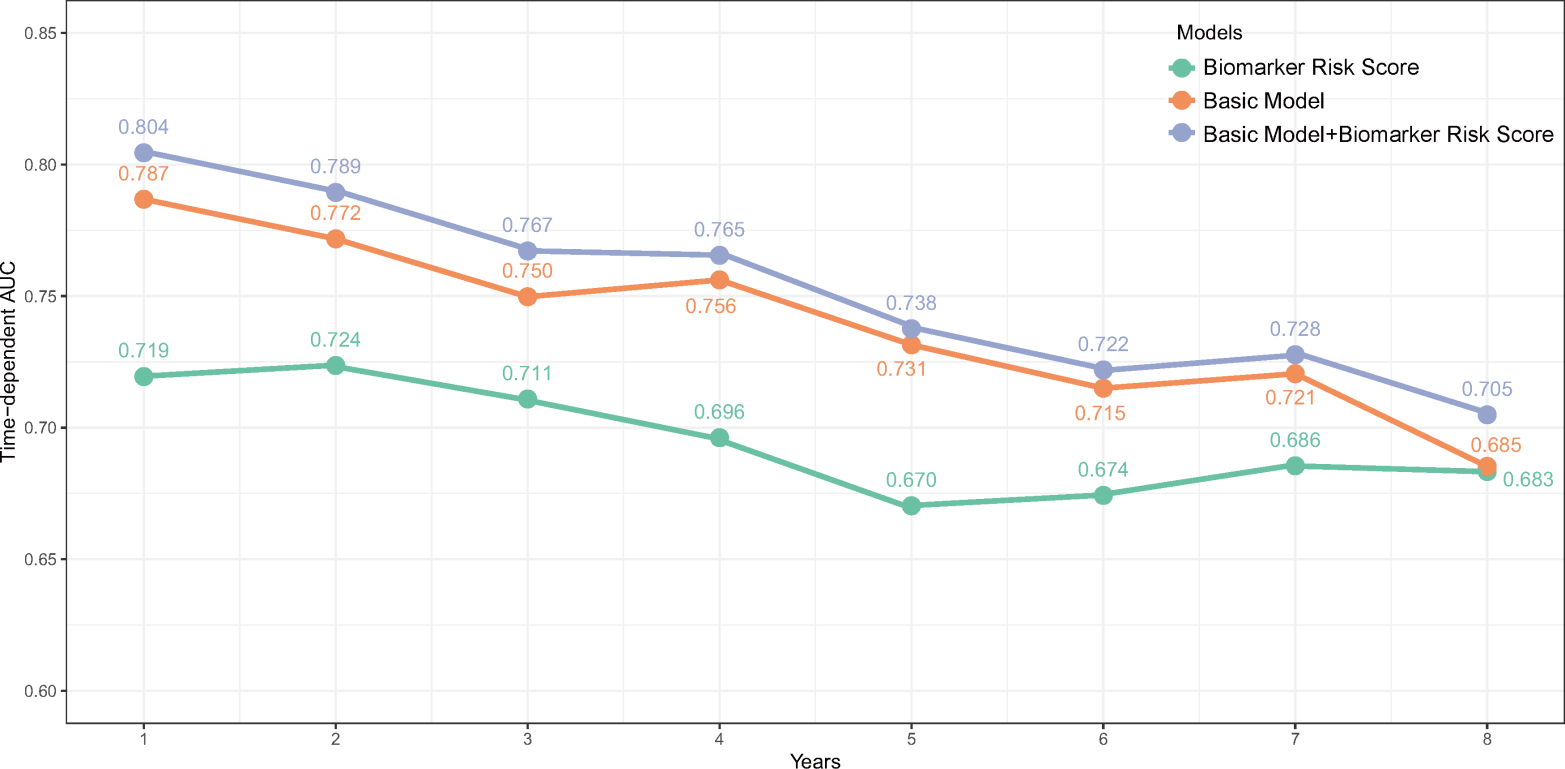
The time-dependent AUC over 8 years of the biomarker risk score, basic model, and the biomarker risk score plus basic model. The basic model was also constructed using elastic net Cox regression incorporating age, gender, SBP, NYHA III/IV, current smoking, DCM, COPD, AF, diabetes, NT-proBNP, creatine, hemoglobin, LDL-C, therapy with ACEI/ARB, and beta-blockers.

**Figure 4:**
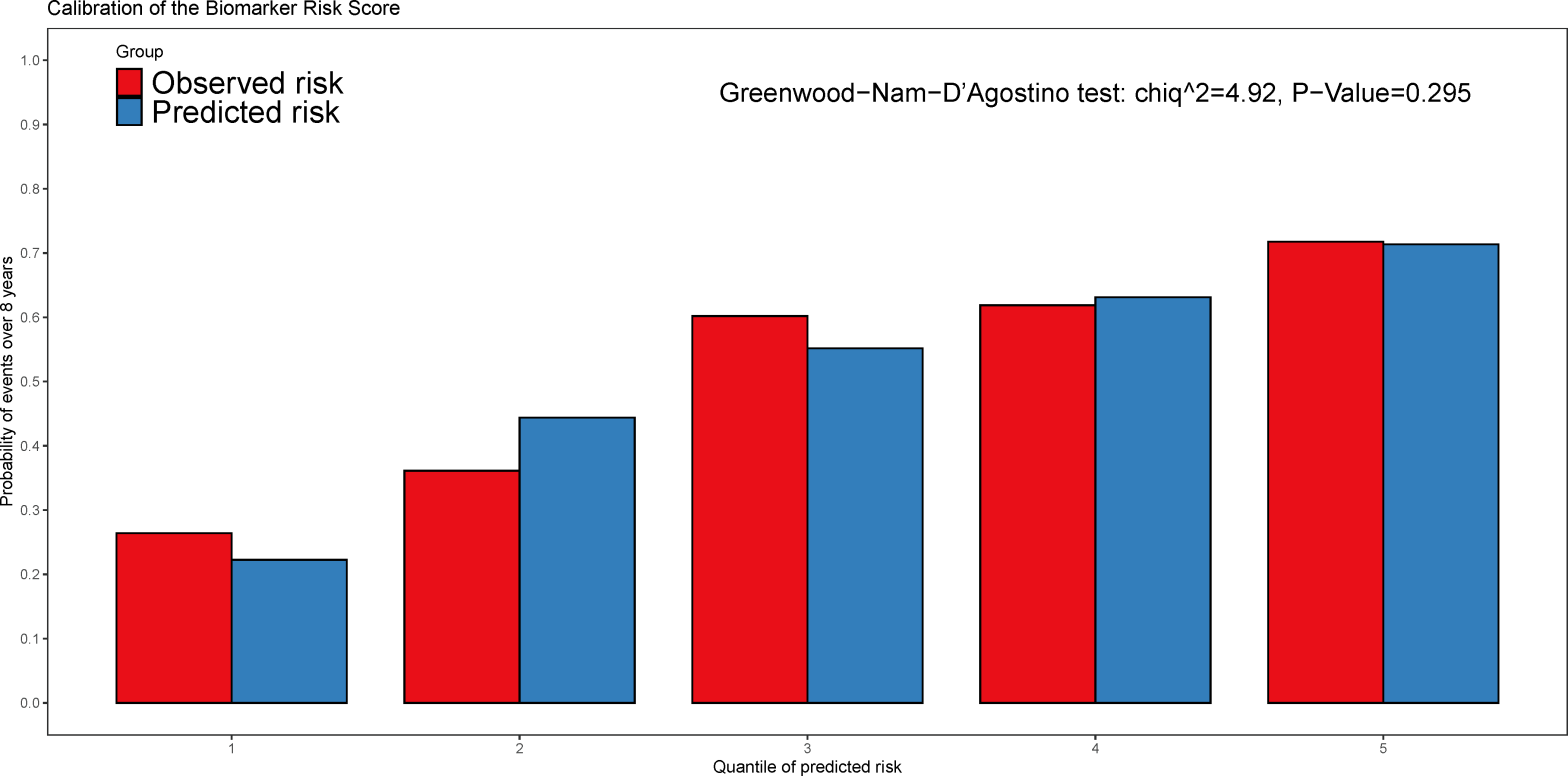
The calibration plot of the biomarker risk score in predicting the all-cause mortality and heart transplantations. The Greenwood-Nam-D’Agostino (GND) test was used for the performance of calibration and *p* > 0.05 indicated the good calibration.

Over a median follow-up period of 2.8 (1.0–4.6) years, 360 patients (28.8%) died, and 76 patients (6.1%) received heart transplants. The time-dependent AUC for the biomarker risk score was 0.720 at 1 year, 0.712 at 3 years, 0.671 at 5 years, and 0.684 at 8 years, as depicted in **Figure 2**. When combining the biomarker risk score with the basic model, the time-dependent AUC at 1 year, 3 years, 5 years, and 8 years was 0.811, 0.780, 0.756, and 0.719, respectively. Moreover, the biomarker risk score exhibited consistent discrimination across cross-validation over 1 to 8 years, with mean time-dependent AUCs ranging from 0.670 to 0.724 for biomarker risk score and 0.705 to 0.804 for the basic model with biomarker risk score added, as shown in **Figure 3**. The addition of the biomarker risk score to the basic model improved the model’s *C*-index from 0.746 to 0.758, with a Δ*C*-index of 0.012 and 95% CI from bootstrapping of 0.003 to 0.018, as demonstrated in **Table 2**. The calibration plot in **Figure 4** indicates that the biomarker risk score was well-calibrated, with a non-significant *p*-value of the GND test (*χ* ^2 = ^4.92, *p* = 0.295). Furthermore, compared to the basic model, the model including the biomarker risk score significantly improved reclassification (IDI, 2.3% [0.7%–4.9%], *p* < 0.001; NRI, 17.3% [6.4%–32.3%], *p* = 0.007).

**Table 2:** Discrimination and reclassification of adding the biomarker risk score to the basic model in predicting prognosis.

	Δ*C*-index	IDI	*p* for IDI	NRI	*p* for NRI
CAR	0.004 (−0.001 to 0.007)	0.011 (0.001–0.028)	0.02	0.099 (−0.019 to 0.191)	0.073
RDW-SD	0.011 (0.002–0.017)	0.016 (0.001–0.038)	0.04	0.144 (0.050–0.284)	0.013
Biomarker Risk Score	0.012 (0.003–0.018)	0.023 (0.007–0.049)	<0.001	0.173 (0.064–0.323)	0.007

The 95% confidential interval (CI) of the ΔC-index was calculated in 1,000 bootstrap samples. The continuous net reclassification improvement (NRI) and integrated discrimination improvement (IDI) analyses at 8 years. CAR: hsCRP-to-albumin ratio; RDW-SD, red blood cell distribution width-standard deviation.

### 3.4 The independent association between the biomarker risk score and the outcome of patients with NIHF

**Figure 5:**
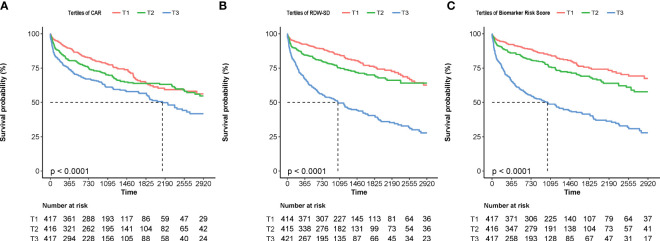
The Kaplan–Meier curves of patients stratified by the tertiles of CAR **(A)**, RDW-SD **(B)**, and the biomarker risk score **(C)**. CAR, hsCRP-to-albumin ratio; RDW-SD, red blood cell distribution width-standard deviation.

The Kaplan–Meier curves show that higher levels of the biomarker risk score were associated with poor prognosis in patients with NIHF when the study population was stratified into groups based on the tertiles of the biomarker risk score (**Figure 5**). In the multivariable regression, after adjusting for confounders (variables within the basic model), the biomarker risk score was also independently associated with the outcome (**Table 3**). With every 1 score increase in the biomarker risk score, the risk of death or heart transplantation in patients with NIHF is expected to increase by 1.76 times (adjusted HR 1.76, 95% CI 1.49–2.08, *p* < 0.001).

**Table 3:** The association between CAR, RDW-SD, and the biomarker risk score with the outcome at univariable and multivariable Cox regression.

	Unadjusted HR	Unadjusted *p*-value	Adjusted HR	Adjusted *p*-value
CAR	1.34 (1.23–1.46)	<0.001	1.17 (1.07–1.28)	0.001
RDW_SD	1.67 (1.54–1.80)	<0.001	1.31 (1.20–1.44)	<0.001
Biomarker Risk Score	2.81 (2.42–3.26)	<0.001	1.76 (1.49–2.08)	<0.001

The adjusted hazard ratio (HR) and p-value were calculated from a multivariable Cox regression adjusting for age, gender, systolic blood pressure (SBP), New York Heart Association (NYHA) III/IV, current smoking, dilated cardiomyopathy (DCM), chronic obstructive pulmonary disease (COPD), atrial fibrillation (AF), diabetes, N-terminal Pro Brain natriuretic peptide (NT-proBNP), serum creatine (Scr), hemoglobin, low-density lipoprotein cholesterol (LDL-C), therapy with angiotensin-converting enzyme inhibitor/angiotensin receptor blocker (ACEI/ARB), and beta-blockers. CAR: hsCRP-to-albumin ratio; RDW-SD, red blood cell distribution width-standard deviation.

## 4 Discussion

In this study, we utilized a machine learning-based elastic net Cox regression for variable selection from easily obtainable inflammatory biomarkers in clinical settings. Ultimately, we constructed a biomarker risk score based on CAR and RDW-SD, and repeated cross-validation demonstrated the high stability of this model. Importantly, a well-calibrated biomarker risk score can improve prognostic prediction in patients with NIHF by increasing discrimination and reclassification performance for all-cause mortality and heart transplantation. The biomarker risk score was also independently associated with adverse outcomes in multivariable regression, suggesting that it can identify high-risk patients and screen potential candidates for inflammation-targeted therapy.

A lot of studies have proved that cell death is a clear trigger of inflammation, which contributes to ischemic HF following MI (16, 17). However, the inflammation observed in NIHF is not initially related to cell death. In contrast to MI, there is modest neutrophil recruitment in the pressure overload heart, which is consistent with the deficiency of cardiomyocyte death. However, the transverse aortic constriction-induced pressure overload heart model shows an increase in F4/80 positive macrophages (3, 18). Research suggests that cardiomyocytes are the primary sites where genes related to inflammation are expressed in response to non-ischemic stressors, including pressure overload, isoproterenol, and angiotensin II (AngII). The Ca2+/calmodulin regulated kinase (CaMKIIδ) activation is the underlying mechanism that triggers cardiac inflammation in non-ischemic stimuli (3, 19). The distinctive inflammatory response and mechanisms of NIHF imply the necessity of studying specific inflammatory biomarkers in HF patients with non-ischemic etiologies.

A previous study by Zhu et al. included 538 patients with acute heart failure (37.9% ischemic HF) and determined CRP, RDW, and NLR as predictors within an inflammatory prognostic score based on the optimal cutoff of 12 inflammatory biomarkers and LASSO analysis (14). However, this study did not exclude patients with infection or acute coronary syndrome during hospitalization; thus, the results may have been affected by these acute inflammatory states. Another study showed that the Pan-Immune-Inflammation Value, calculated by components of complete blood cell counts, is a better prognostic predictor in ST-segment elevation MI patients (20). Nevertheless, there is still a lack of research to establish an inflammation score and thoroughly evaluate its discrimination, calibration, and reclassification performance in patients with NIHF. Our study focuses on NIHF populations without infection or systemic diseases and included 21 inflammatory biomarkers. Therefore, the ultimately screened inflammatory biomarkers (CAR and RDW-SD) may more accurately reflect the damage and repair caused by chronic inflammatory response of cardiomyocytes themselves.

Previous studies have confirmed the prognostic role of the CRP/hsCRP-to-albumin ratio in various diseases (12, 21). In our study, we defined CAR as the ratio of hsCRP to albumin, based on prior research confirming hsCRP as being more strongly associated with cardiovascular disease prognosis than CRP (22). Furthermore, the relationship between hsCRP and the prognosis of HF patients is independent of ejection fraction or etiology (9). Hypoalbuminemia, which is frequently observed in patients with HF, is likely linked to inflammatory states and malnutrition (23). Based on Frank–Starling’s law, a decrease in plasma oncotic pressure resulting from hypoalbuminemia leads to fluid movement from the blood vessels to the tissues, causing cardiogenic pulmonary edema and worsening the prognosis of HF patients (24). Our study identified CAR as a predictor through repeated elastic net regression, rather than hsCRP or albumin alone. Additionally, even after adjusting for factors including NT-ProBNP, CAR remained associated with prognosis. Therefore, we speculate that an increase in the hsCRP-to-albumin ratio may better reflect a patient’s systemic inflammatory state and disease severity than a single indicator. However, the NRI of CAR was not statistically significant (*p* = 0.073), indicating that we need to combine other inflammatory indicators to construct a risk score and improve reclassification.

In addition to CAR, another 100% selected variable in 1,000 times cross-validation was RDW-SD, which reflects the variability of circulating red blood cell size. Studies have shown that RDW is currently considered a marker of chronic inflammation, and there is a significant correlation between RDW and inflammatory parameters (25, 26). As a result of the activation of both cell- and cytokine-mediated inflammatory pathways in HF, the inflammation can cause the release of premature erythrocytes and impair bone marrow function, which leads to an increase in the heterogeneity of red blood cells and the rise of RDW (27). Furthermore, abnormalities in iron metabolization, renal function, and nutrition have also been involved in the pathophysiology of RDW increase in HF patients (28). Although these mechanisms interact and jointly participate in the occurrence and development of diseases, the inflammatory mechanism is at the center of worsening prognosis. Malnutrition, anemia and some other conditions reflected by RDW in HF patients all lead to chronic inflammation of the body, releasing pro-inflammatory cytokines and exacerbating the damage of the heart.

Our previous research found that RDW is an independent predictor of mortality among HF patients across all clinical subtypes (29). Other studies have also shown that RDW can predict long-term outcomes regardless of anemia status in HF patients and as a marker of impaired exercise tolerance in patients with chronic HF (30, 31). Moreover, the RDW-to-albumin ratio (RAR) has been identified as an innovative biomarker of inflammation in HF (32), which is similar to the variable screening results of this study. While RAR was also included as a candidate inflammatory biomarker, the machine learning process ultimately selected RDW-SD, defined as the standard deviation of erythrocyte volumes, as another final predictive factor. Another study also proposed that future research investigating the prognostic value of RDW is expected to concentrate on RDW-SD to eliminate the influence of MCV on RDW (33). In this study, the independent correlation between RDW-SD and prognosis, as well as its good discrimination and reclassification in prognosis prediction, confirmed its effectiveness as an inflammatory predictor. The incorporation of CAR and RDW-SD into a biomarker risk score further enhanced risk stratification beyond the individual biomarker, potentially leading to precise therapeutic interventions targeting the inflammation pathways of patients with NIHF.

This study had several limitations. First, because this study was retrospective, there may be selection bias and potential confounding factors that were not fully accounted for. Secondly, the number of patients who underwent dynamic monitoring of inflammatory biomarkers during follow-up was low, which prevented the analysis of any association between changes in biomarkers and patient prognosis. Thirdly, the study only looked at 21 easily obtainable inflammatory biomarkers, and did not investigate newer, more specific biomarkers, such as those in the interleukin family. Lastly, the inflammatory predictive model established in this study has yet to be validated by an external cohort; hence, caution should be exercised in generalizing its results, and further validation is needed.

## 5 Conclusions

In this study, we developed a biomarker risk score based on two specific biomarkers, CAR and RDW-SD, which were selected from a group of 21 commonly used inflammatory biomarkers using a machine learning approach. The biomarker risk score significantly improved the accuracy of prognostic prediction in patients with NIHF by increasing discrimination and reclassification performance, indicating that it may be a valuable tool for identifying high-risk patients and screening candidates for inflammation-targeted therapy.

## Data availability statement

The raw data supporting the conclusions of this article will be made available by the authors, without undue reservation.

## Ethics statement

The studies involving human participants were reviewed and approved by The Ethics Committee of Fuwai Hospital (Approval numbers 2014-501). The patients/participants provided their written informed consent to participate in this study.

## Author contributions

Conceptualization, JF; methodology, JF; software, JF; formal analysis, JF; investigation, YZ and JZ; data curation, JF, BH, YW, JW, JG, LH, and XL; writing—original draft preparation, JF; writing—review and editing, BH, YW, JW, JG, LH, and XL; supervision, YZ and JZ; project administration, YZ and JZ; funding acquisition, JZ. All authors contributed to the article and approved the submitted version.
