# Supplementary material for: Incorporating inflammatory biomarkers into a prognostic risk score in patients with non-ischemic heart failure: a machine learning approach
Source: Front Immunol. 2023 Aug 15;14:1228018. doi: 10.3389/fimmu.2023.1228018 (PMC10463734; doi:10.3389/fimmu.2023.1228018)
Supplement: Supplementary file 1 [file DataSheet_1.docx]

Supplementary Material

Incorporating inflammatory biomarkers into a prognostic risk score in patients with non-ischemic heart failure: a machine learning approach

Jiayu Feng^1^, Xuemei Zhao^1^, Boping Huang^1^, Liyan Huang^1^, Yihang Wu^1^, Jing Wang^1^, Jingyuan Guan^1^, Xinqing Li^1^, Yuhui Zhang^1^*, Jian Zhang^1 2^*

*** Correspondence:**Yuhui Zhang: yuhuizhangjoy@163.com
Jian Zhang: fwzhangjian62@126.com

# Supplementary Figures and Tables

# 1.1 Supplementary Tables

**Supplementary Table1: Comparison of 11 derived inflammatory biomarkers between patients with primary outcome and patients without primary outcome**

|  | **Overall** | **Primary outcome (-)** | **Primary outcome (+)** | **P-Value** |
| --- | --- | --- | --- | --- |
| **N** | 1250 | 814 | 436 |  |
| NLR | 2.42 [1.83, 3.56] | 2.31 [1.74, 3.24] | 2.72 [2.01, 4.14] | <0.001 |
| PLR | 112.22 [85.36, 147.41] | 109.41 [85.25, 144.28] | 118.19 [85.65, 155.32] | 0.008 |
| NPR | 0.023 [0.022, 0.030] | 0.022 [0.017, 0.029] | 0.024 [0.019, 0.031] | <0.001 |
| LCR | 0.58 [0.23, 1.30] | 0.71 [0.28, 1.51] | 0.41 [0.17, 0.88] | <0.001 |
| RPR | 0.07 [0.06, 0.10] | 0.07 [0.06, 0.09] | 0.08 [0.06, 0.11] | <0.001 |
| RAR | 0.34 [0.30, 0.39] | 0.32 [0.29, 0.37] | 0.36 [0.32, 0.42] | <0.001 |
| PAR | 4.59 [3.58, 5.82] | 4.72 [3.64, 5.93] | 4.43 [3.44, 5.50] | 0.005 |
| FAR | 0.08 [0.07, 0.10] | 0.08 [0.07, 0.10] | 0.09 [0.07, 0.11] | 0.001 |
| CAR | 0.07 [0.03, 0.17] | 0.06 [0.03, 0.15] | 0.09 [0.04, 0.25] | <0.001 |
| SII | 464.03 [329.25, 683.43] | 454.79 [325.47, 651.97] | 497.03 [334.15, 716.30] | 0.051 |
| PNI | 49.77 [45.40, 54.11] | 50.82 [46.70, 54.84] | 47.98 [43.14, 51.90] | <0.001 |

Values are shown as median [interquartile range].NLR, neutrophil-to-lymphocyte ratio; PLR, platelet-to-lymphocyte; NPR, neutrophil-to-platelet ratio; LCR, lymphocyte-to-hsCRP ratio, RPR, RDW-to-platelet ratio; RAR, RDW-to-albumin ratio; PAR, platelet-to-albumin ratio; FAR, FIB-to-albumin ratio; CAR, hsCRP-to-albumin ratio; SII, systematic inflammatory index (neutrophil* platelet/lymphocyte); and PNI, prognostic nutritional index (albumin+5* lymphocyte).

**Supplementary Table2: Average C-index and average partial likelihood deviance of** **models constructed by variables with frequencies 100%, >95% and >90% in 1000 cross-validation**

|  | **Mean C-index** | **Mean Partial-likelihood-deviance** |
| --- | --- | --- |
| **Model1 (100% presented variables)** | 0.680 | 825.71 |
| **Model2 (>95% presented variables)** | 0.679 | 824.87 |
| **Model3 (>90% presented variables)** | 0.683 | 824.26 |

# 1.2 Supplementary Figures


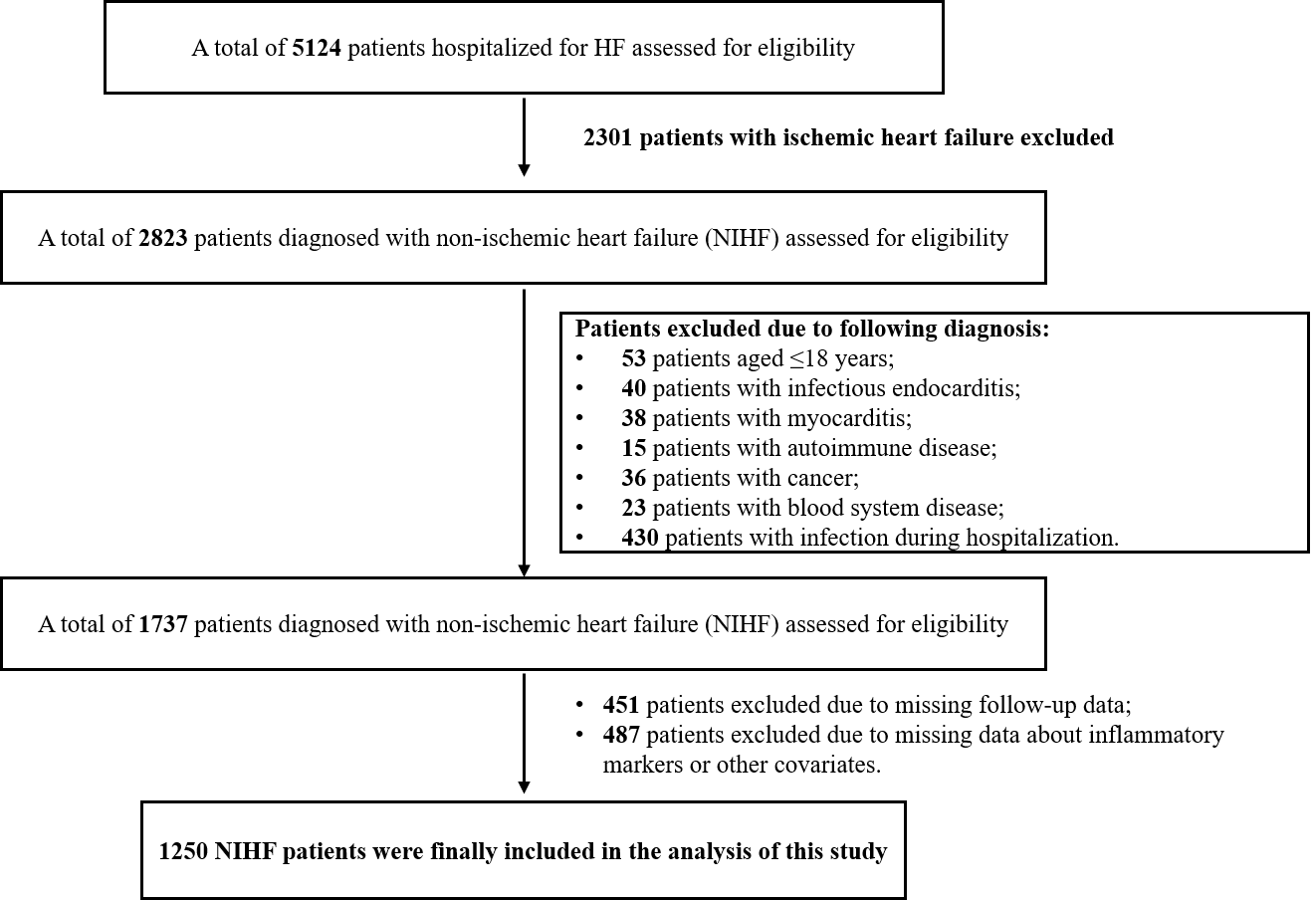


**Supplementary Figure1: The flowchart of this study**


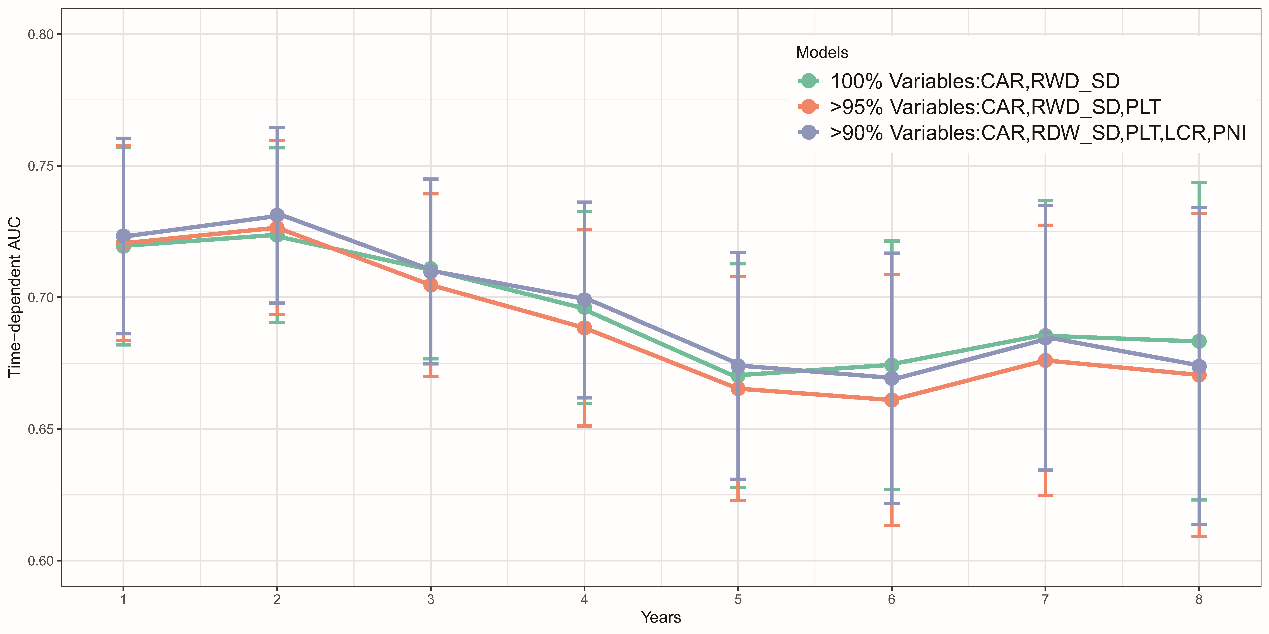


**Supplementary Figure2**: **Mean** **time-dependent AUC for the models constructed by variables with frequencies 100%, >95% and >90% in 1000 cross-validation**
